# The Oldest Anatomically Modern Humans from Far Southeast Europe: Direct Dating, Culture and Behavior

**DOI:** 10.1371/journal.pone.0020834

**Published:** 2011-06-17

**Authors:** Sandrine Prat, Stéphane C. Péan, Laurent Crépin, Dorothée G. Drucker, Simon J. Puaud, Hélène Valladas, Martina Lázničková-Galetová, Johannes van der Plicht, Alexander Yanevich

**Affiliations:** 1 Laboratoire Dynamique de l'Evolution Humaine/UPR 2147, CNRS, Paris, France; 2 Département de Préhistoire/UMR 7194, MNHN-CNRS, Paris, France; 3 Department of Geosciences, University of Tübingen, Tübingen, Germany; 4 Laboratoire des Sciences du Climat et de l'Environnement/IPSL, CEA-CNRS-UVSQ, Gif-sur-Yvette, France; 5 Anthropos Institute/GAČR P405-10-1710, Moravian Museum, Brno, Czech Republic; 6 Center for Isotope Research, Groningen University, Groningen, The Netherlands; 7 Faculty of Archaeology, Leiden University, Leiden, The Netherlands; 8 Institute of Archaeology, National Ukrainian Academy of Sciences, Kyiv, Ukraine; Illinois State University, United States of America

## Abstract

**Background:**

Anatomically Modern Humans (AMHs) are known to have spread across Europe during the period coinciding with the Middle to Upper Paleolithic transition. Whereas their dispersal into Western Europe is relatively well established, evidence of an early settlement of Eastern Europe by modern humans are comparatively scarce.

**Methodology/Principal Finding:**

Based on a multidisciplinary approach for the study of human and faunal remains, we describe here the oldest AMH remains from the extreme southeast Europe, in conjunction with their associated cultural and paleoecological background. We applied taxonomy, paleoecology, and taphonomy combined with geomorphology, stratigraphy, archeology and radiocarbon dating. More than 160 human bone remains have been discovered. They originate from a well documented Upper Paleolithic archeological layer (Gravettian cultural tradition) from the site of Buran-Kaya III located in Crimea (Ukraine). The combination of non-metric dental traits and the morphology of the occipital bones allow us to attribute the human remains to Anatomically Modern Humans. A set of human and faunal remains from this layer has been radiocarbon dated by Accelerator Mass Spectrometry. The direct-dating results of human bone establish a secure presence of AMHs at 31,900+240/−220 BP in this region. They are the oldest direct evidence of the presence of AMHs in a well documented archeological context. Based on taphonomical observations (cut marks and distribution of skeletal elements), they represent the oldest Upper Paleolithic modern humans from Eastern Europe, showing post-mortem treatment of the dead as well.

**Conclusion/Significance:**

These findings are essential for the debate on the spread of modern humans in Europe during the Upper Paleolithic, as well as their cultural behaviors.

## Introduction

The timing of the appearance of early Anatomically Modern Humans (AMHs) in Europe, their hypothesized biological and cultural interactions by replacement or admixture with local populations (Neanderthals or other hominins [1], [2]), and their association with Upper Paleolithic industries are issues subject to continuous debate [3]–[14] despite the increasing number of human fossils, archeological sites and new dates from previously discovered sites.

**Figure 1:**
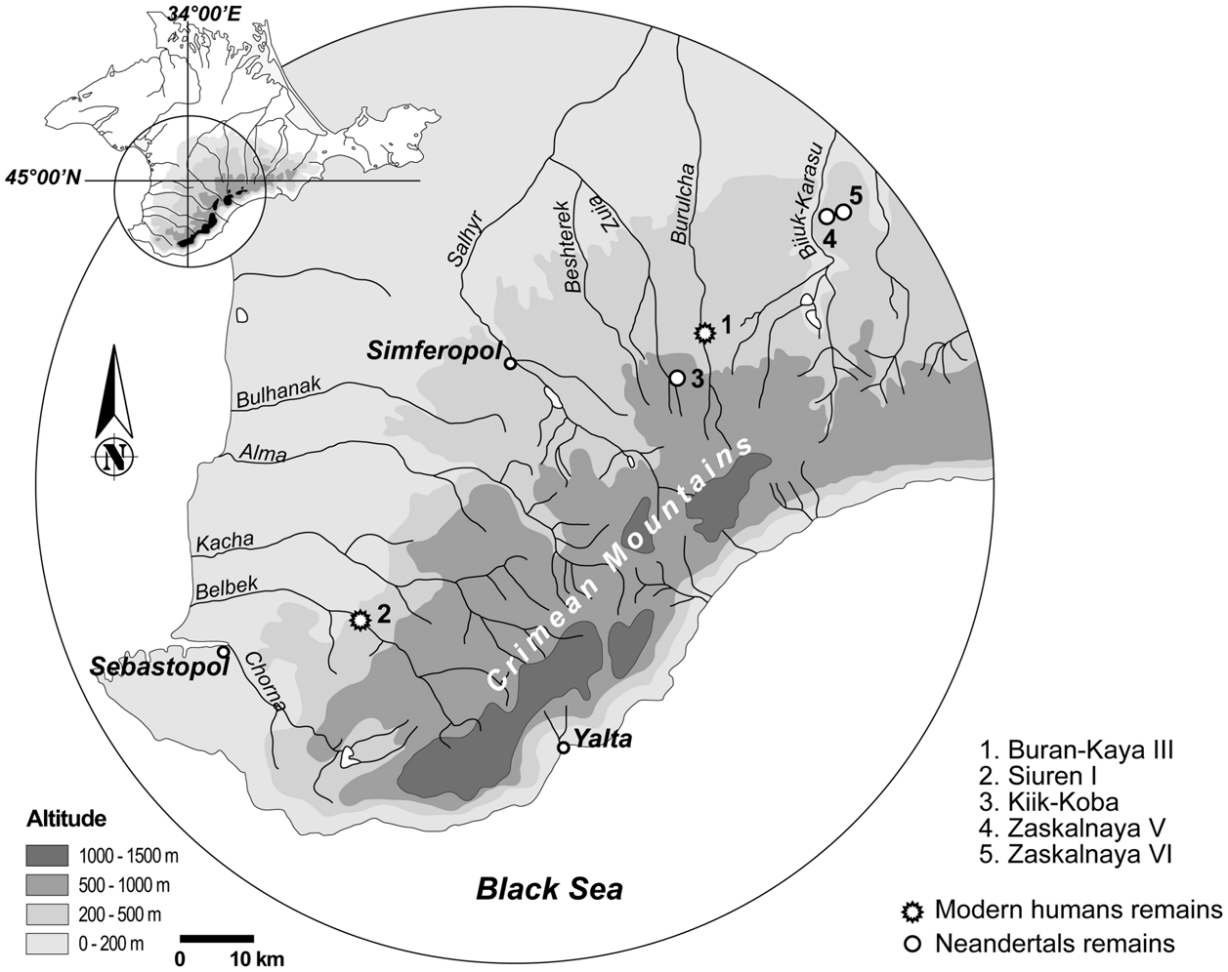
Map of Crimea with the location of Buran Kaya III, Siuren I, Kiik Koba, Zaskal'naya V and VI (modified after [**6]**, [23]–[25****]).

Whereas the arrival and dispersal of modern humans into Western Europe is relatively well established, data related to their early settlement in Eastern Europe is relatively scarce [15], [16]. Buran-Kaya III and Siuren I [17] in Crimea (Ukraine) are the only sites in the northern Black Sea region where a Middle to Upper Paleolithic archeological sequence is well preserved. Crimea is particularly rich in Middle Paleolithic industries and Neanderthal remains (e.g. Kiik Koba (Kiik Koba 1 and 2 [18]–[20]), Zaskal'naya V and VI [21]) (Figure 1). However, with the exception of one molar discovered in Unit G from Siuren I [22], dated to 30–28 ka BP [11], no Upper Paleolithic AMH remains have been reported in this region.

**Figure 2:**
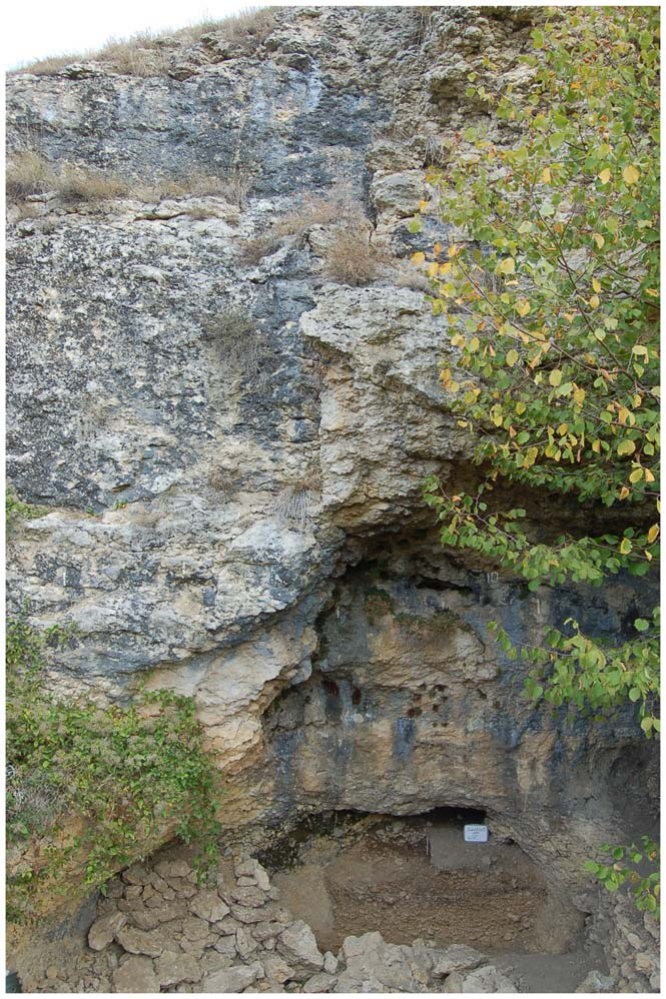
General view of Buran-Kaya III rock-shelter.

We report here the discovery and subsequent first scientific investigation of the human remains from the Buran-Kaya III site. This shelter-cave, overhanging the Burulcha river in the Belogorsk region (N45°00′10.5′′; E34°24′16.3′′) (Figure 2), was discovered in 1990 by A. Yanevich and excavated up to 2001 by a team under the direction of A. Yanevich and A. Marks, with the participation of V. Chabai, Y. Demidenko, K. Monigal, M. Otte and Y. Yamada. Nineteen cultural layers have been identified spanning a period stretching from the Middle Paleolithic to the Middle Ages [26]–[28]. The site was reopened for excavation in 2009 under the direction of A. Yanevich and S. Péan. The excavation and detailed analyses of the human and faunal remains are still continuing.

**Figure 3:**
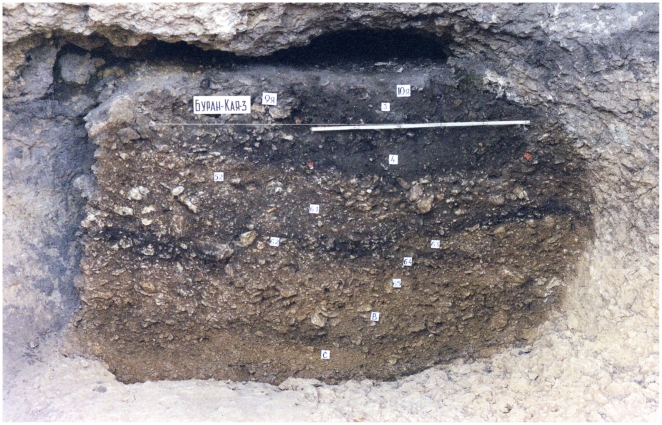
General view of the stratigraphy in Buran-Kaya III. Picture at the end of the 2001 field season.

**Figure 4:**
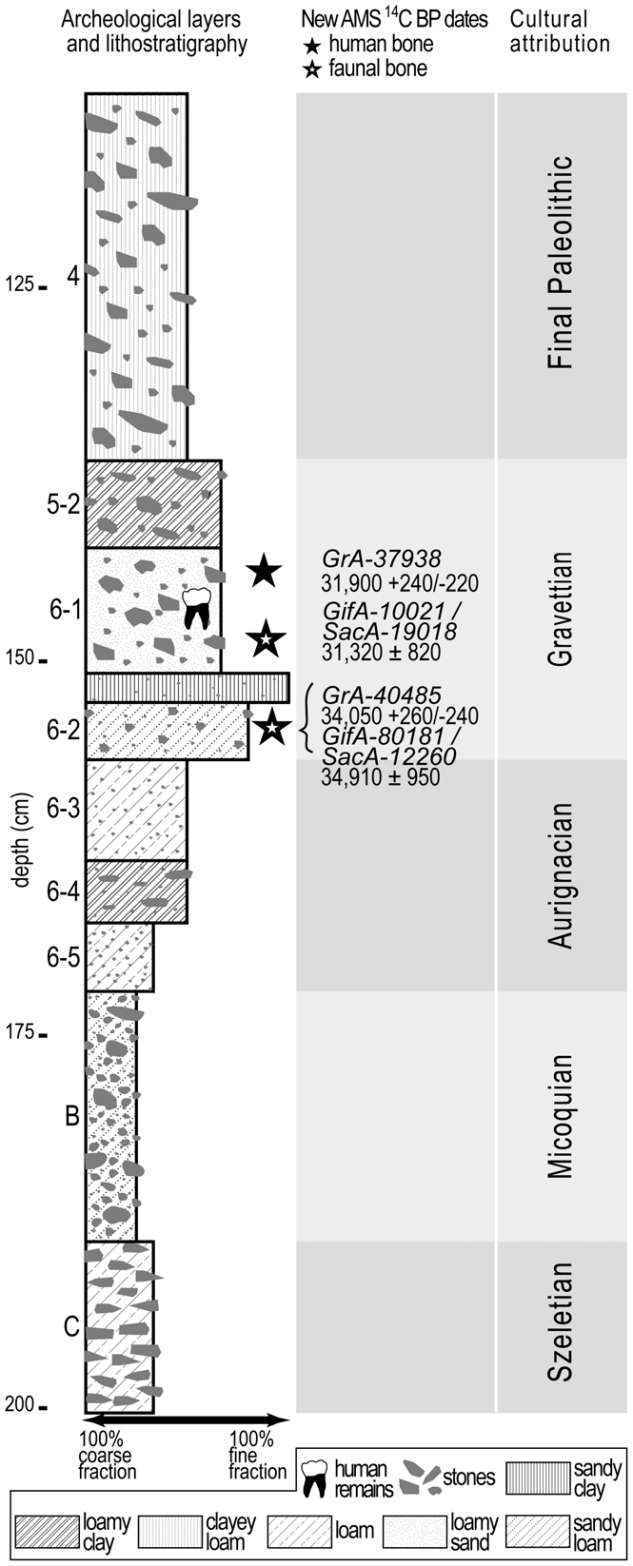
Stratigraphic log, AMS radiocarbon dates and cultural attributions of Buran-Kaya III.

The 2001 and 2009–2010 excavations were focused on nine archeological layers that generated Middle and Upper Paleolithic artefacts: Szeletian (layer C), Micoquian (layer B), Aurignacian (layers 6-5, 6-4, 6-3), Gravettian (layers 6-2, 6-1, 5-2) and Final Upper Paleolithic (layer 4) (Figure 3, Figure 4). A multidisciplinary approach was used to study the following layers: all layers [29], [30], layer C [31]–[33], layer B [34]–[39], layers 6-2, 6-1 and 5-2 [40], [41]. This research demonstrates that Buran-Kaya III is a key site for the understanding of the Middle to Upper Paleolithic transition since it is characterized by the peculiar presence of a Middle Paleolithic layer (Micoquian) situated between two Early Upper Paleolithic layers (Szeletian and Aurignacian).

**Figure 5:**
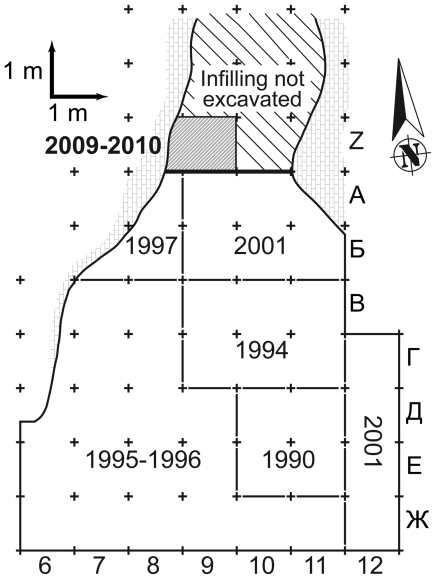
Excavation map of Buran-Kaya III. Fieldwork areas since 1994 and the East-West 2009–2010 stratigraphic section (dark line).

In 2001 A. Yanevich discovered 162 human remains in 6 m^2^ (lines A and Б, columns 9, 10 and 11) of Buran-Kaya III (Figure 5). These remains were found in a well-documented Upper Paleolithic stratum (layer 6-1) with no evidence of an intentional burial pit. In the same layer, 11 remains were found in 1994 (squares 9B, 10 B, 11 B and 9Г) and 8 new ones were recently discovered during the 2009 and 2010 field seasons (square 9Z).

**Figure 6:**
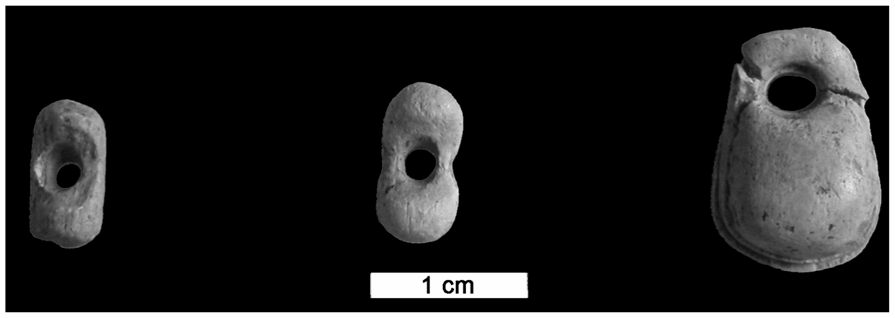
Buran-Kaya III, layer 6-1, body ornaments from mammoth ivory. Scale bar equals 1.0 cm.

**Figure 7:**
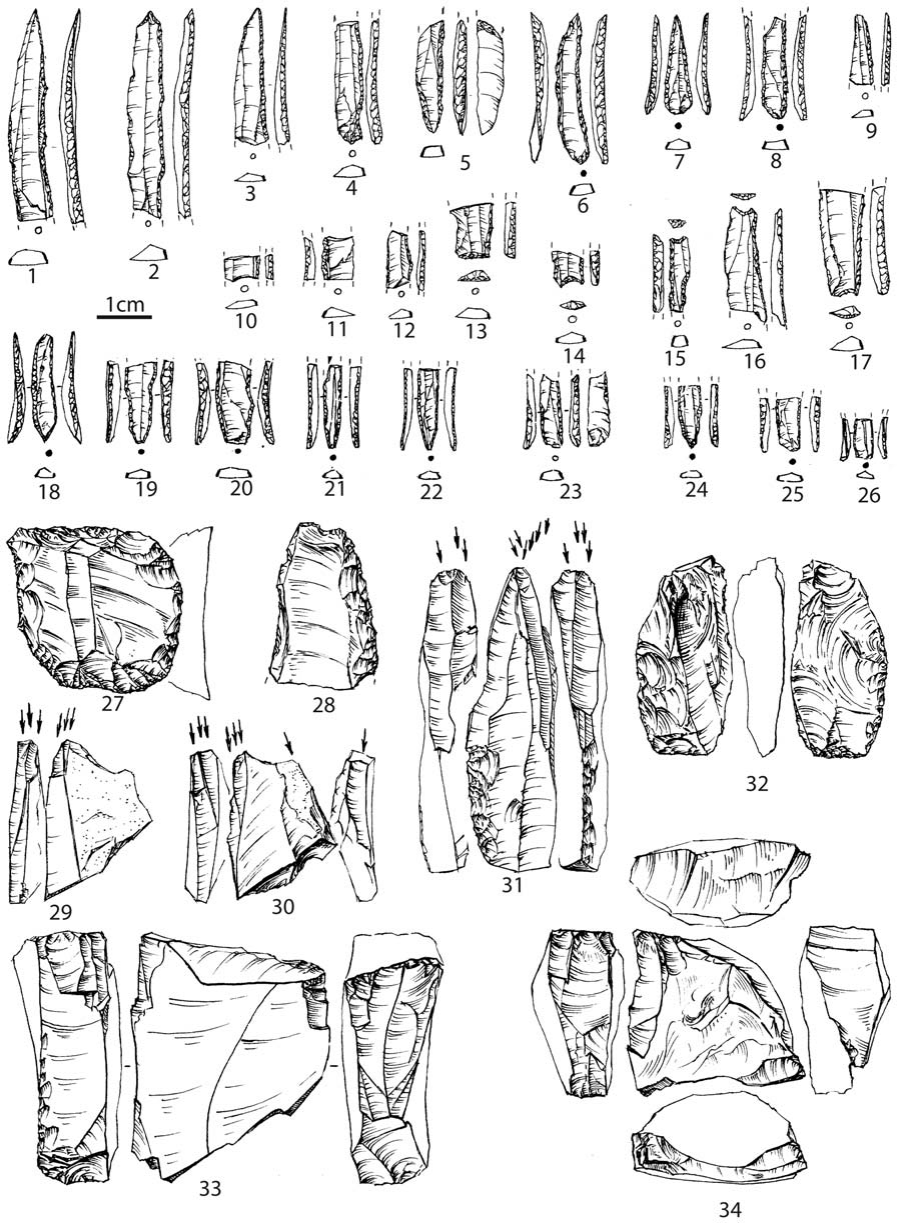
Buran-Kaya III, layer 6-1, lithic industry. 1–26: microliths; 27: end-scraper; 28: blade with retouch; 29–31: burins; 32: *pièces esquillées*; 33–34: cores. Scale bar equals 1.0 cm.

Layer 6-1 has yielded the richest assemblage of lithic and bone industries, body ornaments (Figure 6), ochre fragments, and faunal, human and paleobotanical remains [41] from the site. Lithic artefacts have been attributed to the Gravettian cultural tradition [26], [40] (Figure 7). The bioarcheological features of the layer 6-1 enabled us to investigate the taxonomical attribution of these anthropological finds as well as the relevant cultural behavior in their paleoenvironment context.

## Results and Discussion

### Geomorphological data, paleoenvironmental and archeological contexts

The Buran-Kaya III shelter-cave is a “differential gelifraction balm” [42] due to erosion of the cretaceous limestone banks of the cliffs by frost breaking processes. The sedimentary infilling of this shelter-cave is composed of diamicton layers with angular or slightly rounded autochtonous limestone fragments and a small amount of loamy or sandy matrix. The latter has an aeolian origin but could have been also deposited by water from the overlying plateau. These lithostratigraphic facies suggest that the sequence was formed in a periglacial environment, corresponding to a dry and cold period. However, a thin sandy clay lens between layers 6-2 and 6-1 would indicate more temperate conditions. The results of paleoecological studies, based on pollen [43], microfauna [30], large mammals (see paragraph faunal assemblage) and sediments, indicate an open environment characterised by a reduced tree cover with a cold and arid climate that progressively become more severe from the bottom to the top of layer 6-1.

The abundant lithic assemblage is made up of 23,027 artefacts of which 3.5% are retouched tools, including end-scrapers, burins and numerous backed microliths (84.4% of retouched tools) attributed to the Gravettian tradition. The bone industry consists of pointed tool fragments (projectile points, awls) as well as manufacturing debris from the *in situ* production of points or needles. The personal ornaments are represented by 5 mammoth ivory beads, 1 engraved plate made of mammoth ivory and 35 marine and fresh water perforated shells (with natural and human-made perforations). The lack of blank, roughed-out, waste products or mammoth remains suggest off-site production and subsequent import of the finished mammoth ivory ornaments.

### Stable isotopes and radiocarbon dating

A human parietal fragment from layer 6-1 was radiocarbon dated at 31,900+240/−220^ 14^C BP (GrA-37938) by Accelerator Mass Spectrometry (AMS) (Table 1). In addition, the AMS radiocarbon dating of a red deer metacarpal from layer 6-1 yielded an age of 31,320±820 ^14^C BP (GifA-10021/SacA-19018). Furthermore, one horse metatarsal from layer 6-2, was dated by the two different laboratories independently, and provided comparable results: 34,050+260/−240 ^14^C BP (GrA-40485) and 34,910±950 ^14^C BP (GifA-80181/SacA-12260). The chemical quality parameters for the fossil bones (organic content, C/N ratio and stable isotope ratios) are also shown in Table 1. These four new dates are chemically reliable and provide a chronology consistent with the stratigraphic sequence of the samples.

**Table 1:** Radiocarbon and stable isotopes results for the human and faunal remains from Gravettian layers 6-1 and 6-2 of Buran-Kaya III, Crimea.

	*Homo sapiens* (parietal)		*Cervus elaphus* (metacarpal)	*Equus* sp. (metatarsal)	
Laboratory number	GrA-37938		GifA-10021/SacA-19018	GrA-40485	GifA-80181/SacA-12260
Layer	6-1		6-1	6-2	
Radiocarbon age (AMS ^14^C years BP)	31,900+240/-220		31,320±820	34,050+260/-240	34,910±950
“Calendar” age (cal BP age) based on IntCal09 [44]. 95.4% (2 σ) Cal age ranges	35,488–35,980	36,163–36,912	34,474–37,986	38,408–40,062	37,599–41,745
Relative area under distribution	0.207	0.793	1.0	1.0	1.0
C (%)	43.2		43	32.2	
N (%)	15.3		15	11.5	
C/N *	3.3		3.3	3.3	
δ^13^C (‰)	−19.4		−19.1	−19.2	
δ^15^N (‰)	15.4		7.9	9.0	

*C/N corresponds to the atomic ratio calculated as follows: (C content/N content)*(14/12). The dates were calibrated using the sofware calibREV6.0.0 based on IntCAl09 calibration dataset [44].

**Figure 8:**
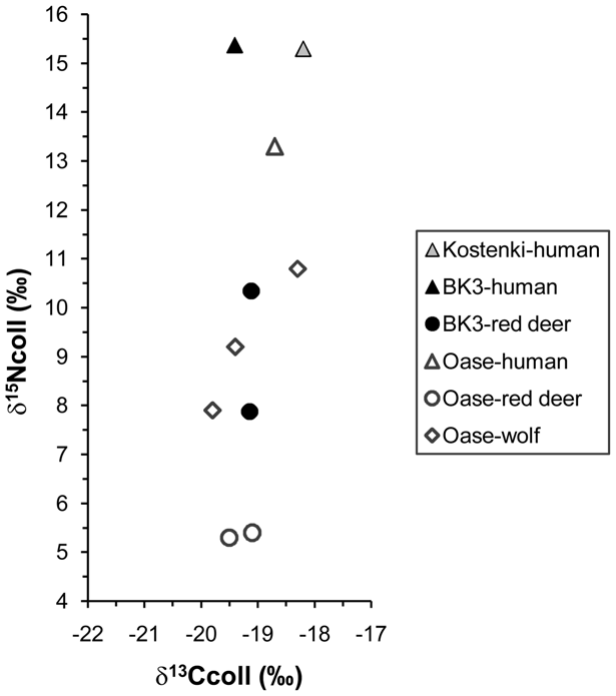
Comparative isotopic data (Buran-Kaya III, Kostenki 1 and Oase). Collagen δ^13^C and δ^15^N values of human and fauna remains from the layer 6-1 of Buran-Kaya III (ca. 32,000-30,500 BP; this work), of human bone from Kostenki 1 (ca. 31,500–34,000 BP [47]) and from layer 1 and 2 of Oase (ca. 58,000-45,000 BP for the red deer and wolves, ca. 34,000–36,000 BP for the human mandible [48], [49]).

The C/N atomic ratio (3.3) of the dated human fragment is within the acceptable range of 2.9–3.6, expected for well-preserved collagen [45],[46]. The δ^15^N value on dated collagen of the Buran-Kaya III human bone (15.4‰) is similar to the one measured on the Kostenki 8 human fossil from the site of Kostenki 1 (15.3‰), a result interpreted as reflecting more than 50% of the diet originating from freshwater-derived protein [47]. Similarly, high consumption of fish is considered the likely explanation for the Oase 1 human mandible's high δ^15^N value (13.3‰ [48]), which is about 8‰ higher than those found for the red deer from the same site (5.3 and 5.4‰ [49]). This exceeds the expected range of 3 to 5‰ between a consumer and its preys (e.g. [50]). Interestingly, if the human δ^15^N value in Buran-Kaya III is about 2‰ higher than the one found in Oase, the associated red deer are about 4‰ higher in Buran-Kaya III compared to Oase. As a result of those high ^15^N abundances, the red deer of layer 6-1 have a similar isotopic signature to those of the wolves of Oase (Figure 8). Thus, we observe different δ^15^N values for terrestrial ecosystems in Eastern Europe during the early Upper Paleolithic. The higher ^15^N amounts found in fauna from Buran-Kaya III in Crimea compared to Oase in Romania could be explained by environmental differences between both regions. However, we should also consider the hypothesis of a chronological increase in δ^15^N values between Oase (layer 1 and 2) where red deer, as well as wolves, were older than ca. 45,000 BP [48] and Buran-Kaya III (layer 6-1) for which we obtained AMS dates of 30,500–32,000 BP. Such a shift towards higher δ^15^N values in terrestrial food webs was observed around the Middle to Upper Paleolithic transition in western Europe [51]. Analysis of stable isotopes for chronologically and geographically nearby fauna is necessary for thorough assessment of the human diet. The contribution of aquatic resources is needed not only to study the evolution of food patterns, but also to quantify the marine or freshwater reservoir effect for ^14^C dates [52]. Preliminary biogeochemical results on the coeval mammals from layer 6-1 of Buran-Kaya III suggest a diet composed of mainly terrestrial resources and only a moderate consumption of freshwater food for the dated human. A reservoir correction for the radiocarbon dates is not significant considering the measurement uncertainties.

### Faunal assemblage and taphonomical analyses

Layer 6-1 yielded 112,552 faunal remains, of which less than 1.8% were anatomically and taxonomically identified (NISP = 1,625; Total cMNI = 39). The faunal assemblage shows a predominance of saiga antelopes, mostly represented by adult individuals (*Saiga tatarica*, 42% of NISP, cMNI = 15), red and polar foxes (*Vulpes vulpes* and *Alopex lagopus*, 29.5% of NISP, cMNI = 7) and hares (*Lepus* sp., 13% of NISP, cMNI = 5). The other mammals determined are equids (*Equus* sp., 10% of NISP, cMNI = 3*)*, bovines (*Bison* sp. or *Bos* sp., 4% of NISP, cMNI = 2), carnivores (*Canis lupus, Ursus* sp., *Felis silvestris* and Mustelidae, 1% of NISP, cMNI = 5), and cervids (*Cervus elaphus* and *Rangifer tarandus,* 0.5% of NISP, cMNI = 2).

**Figure 9:**
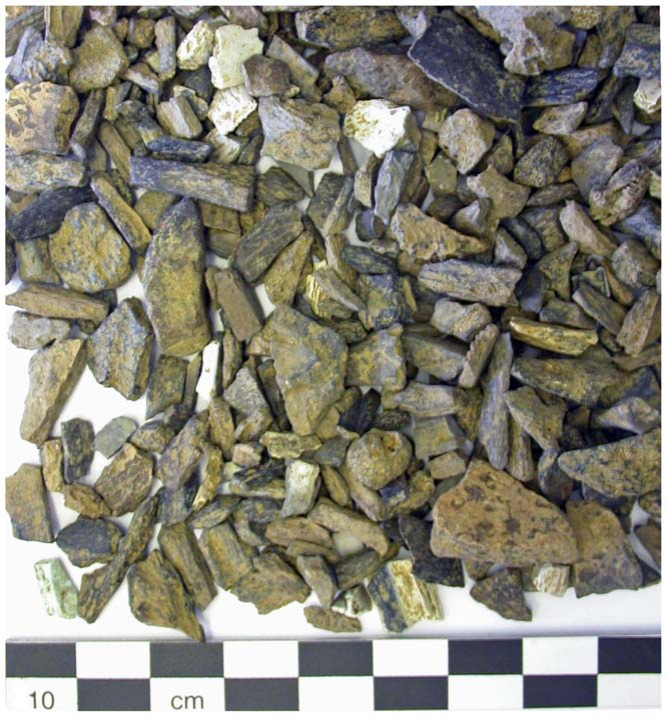
Buran-Kaya III, layer 6-1, burned bones. Scale bar equals 1.0 cm.

The taphonomical analyses of the faunal bones from layer 6-1 show a very high fragmentation rate with less than 10% of the material longer than 2 centimeters. These studies show limited carnivore bone surface modification of mammal bones (tooth marks are only observed on 1% of NISP). Almost all the skeletal elements of saiga antelope, fox and hare are represented but axial and girdle parts are relatively scarce. A high proportion of burned bones were found (25% of total number of remains) (Figure 9), with high range of color diversity (from brown to white). Especially 9.5% of them exhibit a white coloration which indicates calcination. According to an experimental chart [53], the white color reveals an intensity of heat more than 700°C. Ground temperatures higher than 700°C are never reached in natural fires in forest, shrubland and grassland ecosystems [54]. Thus, the burning of the bone material from layer 6-1 is supported to have resulted from human activities rather than natural phenomena.

**Figure 10:**
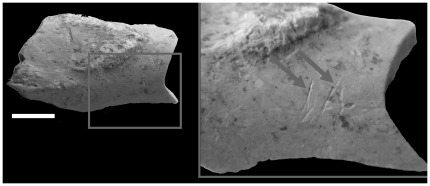
Buran-Kaya III, layer 6-1, cut marks on proximal diaphysis of right humerus from Saiga antelope. Medial view, scale bar equals 1.0 cm.

Cut marks and fracture features due to human activities are observed on 7% of the NISPs of the following 6 taxa: saiga antelope, hare, bovine, horse, fox and wolf (Figure 10). Human modifications of the saiga bone surfaces (on cranial and postcranial remains) mainly reflect disarticulation and defleshing activities, breakage and marrow extraction. Consumption of hares is also evidenced by taphonomical modifications. Foxes exhibit marks of disarticulation and defleshing, and also traces of fur removal on metapodials and tarsals. Saiga antelope represents the main game of the hunter-gatherers in the site.

### Human remains: taxonomic attribution, skeletal distribution and human modification

A total of 162 human remains were discovered in layer 6-1 during the 2001 field season. They were spread over 6 m^2^ of the entire excavated area. These remains consist mostly of highly fragmented cranial parts (n = 117) and teeth (n = 29), whereas the postcranial skeleton (n = 16; MNI = 2, juvenile and adult) is barely represented (7 rib fragments, 1 vertebra and 8 hand phalanx fragments). At least 5 individuals were identified from the dental remains. They belong to 3 different developmental age groups: juvenile (n = 1), sub-adult (n = 2) and adult (n = 2). The human skeletal distribution shows a clear lack of anatomical parts (especially long bones from upper and lower limbs) which are usually preserved. This cannot be explained by differential preservation processes since other mammals are conversely represented by most, if not all, skeletal elements.

**Figure 11:**
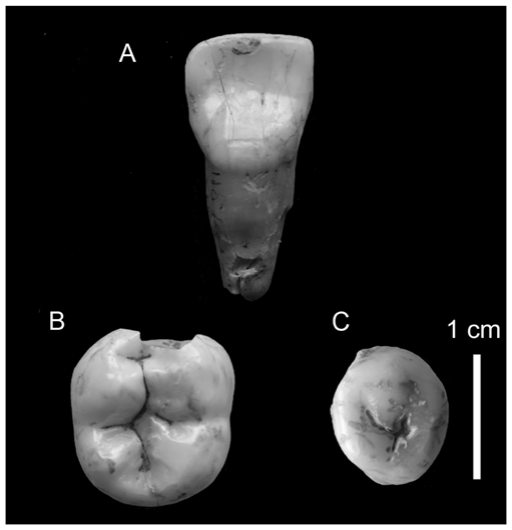
Buran-Kaya III, layer 6-1, human dental remains. A, right first upper incisor, lingual view, B, right lower second molar, occlusal view, C, right lower second premolar, occlusal view. Scale bar equals 1.0 cm.

**Figure 12:**
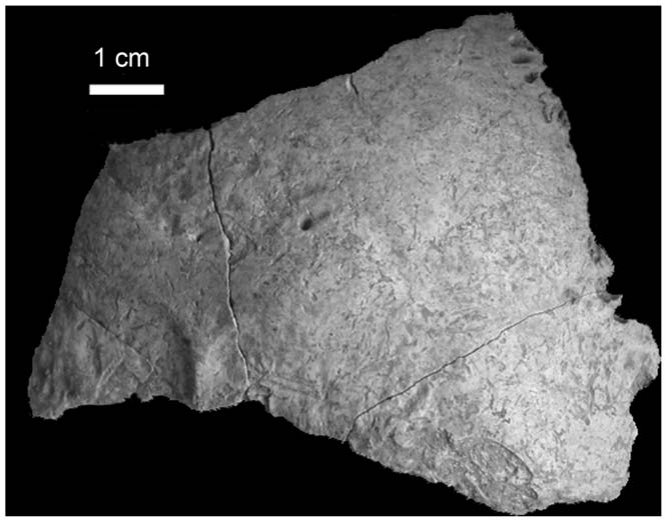
Buran-Kaya III, layer 6-1, occipital bone. Posterior view, scale bar equals 1.0 cm.

The taxonomic attribution of the human remains was made possible thanks to the well-preserved cranial bones and teeth (from both 1994 and 2001). The dental remains exhibit traits that occur more frequently in Anatomically Modern Humans (AMHs) than in Neanderthals; namely, the symmetry observed in the occlusal outline, the lack of a well-developed metaconid and a transverse crest on the lower second premolar, the lack of a well-developed mid-trigonid crest and a large anterior fovea on the lower first and second molars, the lack of shovelling, labial convexity and well-developed lingual tubercles on the upper first and second incisors (Figure 11). The combination of these traits taxonomically distinguishes AMHs from Neanderthals [12], [55]. Furthermore, the occipital bones and especially the specimen BK3-55 (Figure 12) do not show an occipital “bun” and do not present a bilaterally transverse occipital torus nor a suprainiac fossa which are considered as typical Neanderthal features [56]–[58]. Therefore, based on this suite of morphological features, the human remains from Buran-Kaya III can be attributed to Anatomically Modern Humans.

**Figure 13:**
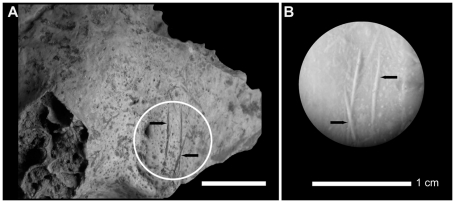
Anthropogenic modifications observed on a right temporal human bone from Buran-Kaya III, layer 6-1. A, location of the cut marks on the exocranial surface (black arrows), lateral view. B, stereomicroscopic image of the mould. Scale bars equal 1.0 cm.

**Figure 14:**
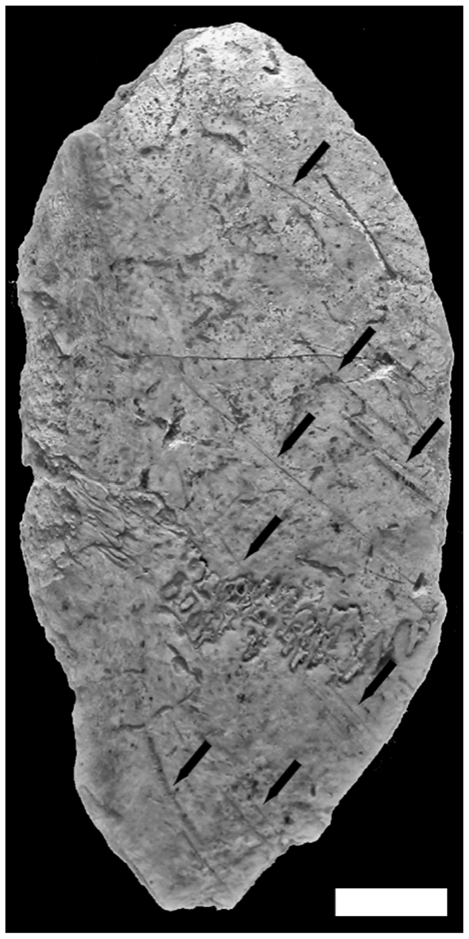
Anthropogenic modifications observed on a left fronto-parietal human bone fragment from Buran-Kaya III, layer 6-1. Superior view, scale bar equals 1.0 cm.

Regarding the human modifications, the presence of cut marks on the exocranial surface of 14 remains, especially on one occipital bone (Figure 12), one temporal bone (Figure 13), one fronto-parietal fragment (Figure 14), was confirmed by analyses under a stereomicroscope and Environmental Scanning Electron Microscope (ESEM). The striations are multiple and parallel. Their morphological features include V-shaped cross section, shoulder and splitting effects. No percussion marks, reflecting breakage and marrow extraction, were observed on cranium and mandible, and no taphonomical marks from carnivores were identified. The observed cut marks, according to their morphology, location (position relative to muscle insertions) and distribution (number and orientation) suggest two different processes: scalping on the fronto-parietal fragment and disarticulation on the occipital and temporal bones.

The taphonomical modifications observed on the Buran-Kaya III early AMHs, along with the over-representation of cranial remains, suggest a post-mortem processing of human body parts, including intentional selection of the skull. Dietary and non-dietary cannibalisms as well as mortuary practices are the most common explanations proposed for anthropic actions on human bodies [59]–[62]. Dietary cannibalism (“the use of humans by humans as food” [63]) is usually described as either survival (“starvation-induced cannibalism” [60]) or gastronomic [60], gustatory or nutritional (“consumption of human flesh not as a starvation avoidance” [61]). In general, evidence for dietary cannibalism is shown by a similar treatment of animal and human remains, in terms of butchering marks, anatomical element representation, marrow extraction, post-processing discard and cooking [63]. Non-dietary cannibalism is notably called either funerary cannibalism (“cannibalism of deceased, usually within-group members in which affectionate motivation is present”[60]) or ritual cannibalism (“consumption of specific body parts not as food source but as part of mortuary ritual” [61]).

**Figure 15:**
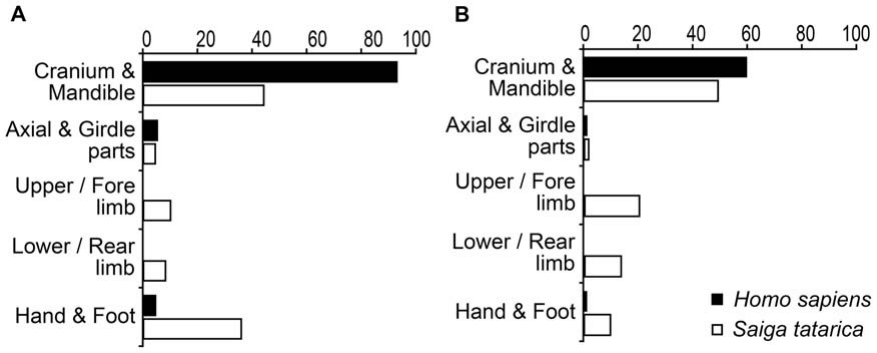
Comparison of the skeletal preservation between *Homo sapiens* and *Saiga tatarica* from Buran-Kaya III, layer 6-1. A, percentage of number of identified specimens (**%**NISP). B, Percentage of element Representation (PR or % survival) defined as PR(i) = (MNE(i)*100)/(N(i)*cMNI).

In Buran-Kaya III, the representation of skeletal elements (%NISP and Percentage of element Representation) of the human and faunal remains (illustrated by saiga antelope, predominant prey) are noticeably different: absence of human upper and lower limbs and over-representation of human skulls compared to post-cranial parts (Figure 15). Conversely, all the skeletal elements of saiga antelope are represented. The percentages of bones with cut marks are also different: cut marks are observed on 9.5% of the human skull remains, 1.3% for saiga antelope; on 0% of the human post-cranial remains, 7.7% for saiga antelope. The modification frequencies are significantly different (chi 2 = 51.65, p<0.05, ddl = 2). Marrow extraction is evidenced only in saiga antelope (mandible, long bones and phalanges). These features suggest that humans and main game of the hunters-gatherers were not processed in a similar way. Thus, the hypothesis of a dietary cannibalism, as defined above [63], does not seem to be supported by evidence based on body treatment. We consider an alternative scenario. The modifications on human remains could be interpreted as a mortuary ritual, either ritual cannibalism or a specific mortuary practice: post-mortem disarticulation processes of corpses for secondary disposal. The primary processing of the deceased body would have occurred in the site, where mainly cranial and dental remains along with a few phalanges, ribs and vertebra were leftover. The remaining parts of the body would have been taken outside the site by humans (at least as far as the excavated part of the site is concerned). Furthermore, the association with imported body ornaments reinforces the hypothesis of symbolic behavior related to the treatment and disposal of the human remains.

Our multidisciplinary research focus of Buran-Kaya III provides a new dataset of early Anatomically Modern Human remains, directly dated by radiocarbon, and securely associated within a well-documented archeological context of the Gravettian cultural tradition.

Our results lead to three major implications for the understanding of the colonization of Europe and related Upper Paleolithic cultural manifestations.

1) The direct date of human bone from Buran-Kaya III (layer 6-1) at 31,900+240−220 BP (GrA-40485) is the oldest one in far southeastern Europe. Buran-Kaya III (layer 6-1) provides one of the oldest direct indications for the presence of Anatomically Modern Humans in Europe and therefore increases the number of European modern human remains directly dated between 40,000 and 29,000 BP. We highlight, in Table 2, the other European Anatomically Modern Human remains directly dated between 40-29 ka BP with a quite secure taxonomical attribution.

**Table 2:** European Anatomically Modern Human remains directly dated between 40-29 ka BP with a quite secure taxonomical attribution.

Site	Country	Specimen No.	Element	^14^C date (BP) and laboratory number
Kostenki 1 (layer III) [47], [64], [65]	Russia	Kostenki 8	Undescribed tibia and fibula	32,600±1,100 (OxA-7073)
Peştera cu Oase [66]	Romania	Oase 1	Mandible	34,290+970/−870 (GrA-22810)
		Oase 1	Mandible	>35,200 (OxA-11711)
Peştera Muierii [67], [68], Baia de Fier	Romania	Peştera Muierii 1	Scapula and tibia	30,150±800 (LuA-5228)
		Peştera Muierii 1	Cranium	29,930±170 (OxA-15529)
		Peştera Muierii 2	Temporal	29,110±190 (OxA-16252)
Peştera Cioclovina Uscată [68]–[70]	Romania	Cioclovina 1	Temporal	29,000±700 (LuA-5229)
		Cioclovina 1	Occipital	28,150±170 (OxA-15527)
Mladeč [71]	Czech Republic	Mladeč 1	Right upper second molar	31,190+400/−390 (VERA-3073)
		Mladeč 2	Left upper third molar	31,320 +410/−390 (VERA-3074)
		Mladeč 8	Left upper second molar	30,680+380/−360 (VERA-3075)
		Mladeč 9a	Right upper canine	31,500+420/−400 (VERA-3076-A)
		Mladeč 9a	Right upper canine	27,370±230 (VERA 3076-B)
		Mladeč 25c	ulna	26,330±170 (VERA-2736)
Obłazowa cave [72]	Poland	Obłazowa	Distal thumb phalanx	31,000±500 (OxA-4586)
La Crouzade [73]	France	La Crouzade VI	Maxilla	30,640±640 (ERL-9415)
Goat's Hole, Paviland [74]	United Kingdom	Paviland 1	Rib	28,870±180 (AF, OxA-16412)
		Paviland 1	Rib	28,400±320 (AG, OxA-16502)
		Paviland 1	Scapula	29,490±210 (AF, OxA-16413)
		Paviland 1	Scapula	28,820±340 (AG, OxA-16503)

2) Layer 6-1 provided lithic artifacts and ivory ornaments securely attributed to the Gravettian cultural tradition [26], [40], with unexpected early ^14^C dates. This association is free from material sedimentary overlap, as shown by the stratigraphic and sedimentologic work conducted during the 2001 and 2009 field seasons. There is a continuity in the Buran-Kaya III stratigraphical sequence from the lower layers (6-5, 6-4, 6-3) that provided lithic elements consistent with the Aurignacian cultural tradition up to the upper layers (6-2, 6-1, 5-2), where lithic industries exhibited features of the Gravettian technocomplex.

Radiocarbon dates of approximatively 31–32 ka BP have been associated mostly with Aurignacian [6], [11], [12], [75], but also with Gravettian settlements: e.g. in Poland (31,000±550 BP (OxA-4586) in Obłazowa cave, layer VIII [72]), Austria (30,500+900/−800 BP (GrN-11193) in Willendorf II, layer 5 [76]), and Ukraine (29,650±1,320 BP in Molodova V, layer IX [77]). Thus, the new Buran-Kaya III ^14^C dates confirm the hypothesis of a very early occurrence of Gravettian settlements prior to 30 ka BP in Eastern Europe [78], [79].

3) These results also shed new light on the symbolic behavior of early Anatomically Modern Humans, in terms of treatment of corpses. To date, two ^14^C dated Aurignacian settlements from Western Europe have yielded early modern human remains bearing cut marks: La Crouzade [73] (31-30 ka BP) and Les Rois [80] (28–30 ka BP, taxonomical attribution of the mandible under discussion) in France. Thus, proven by direct dating, Buran-Kaya III seems to represent the oldest evidence in Europe at 32 ka BP of cut mark actions on modern human corpses, which seem to be related rather to ritual cannibalism or mortuary practices than to dietary cannibalism.

Our findings fill an important gap in the study of modern human settlements in Eastern Europe. The human fossils represent the oldest direct evidence of the presence of modern humans in far southeastern Europe. Our multi-disciplinary studies yield new insights into the subsistence and symbolic behaviors of the earliest european AMHs. In particular, anthropogenic modifications of human bones represent the oldest evidence of postmortem treatments of the dead by early modern humans in Europe.

Specific aspects of the Crimean early Upper Paleolithic lithic industries need to be further investigated, especially in the context of this new radiocarbon dated sequence. Crimea and Eastern Europe represent important regions for a better understanding of the dispersal of early AMHs and the diffusion of Upper Paleolithic cultures with their related symbolic behavior.

## Materials and Methods

In order to study the Upper Paleolithic layers of Buran-Kaya III, we carried out radiocarbon dating, plus geomorphological, stable isotopes, zooarcheological, taphonomical and anthropological analyses.

### Geomorphological analyses

Geomorphological research on the formation of the shelter-cave and studies of its sedimentary infilling were undertaken to better understand the depositional processes. Sections were drawn and deposits were described macroscopically and microscopically in order to distinguish the different sedimentary layers and establish a stratigraphic sequence. Its detailed description takes into account the texture of the sediment matrix (sand, silt, clay), its color according to the charts of Cailleux [81] and Munsell [82], its composition (quartz, limestone) and porosity. The elements of the coarse fraction (larger than 2 mm) were also described in terms of nature, shape and surface preservation. The synthesis of these first results provides information concerning the origin and the mode of sediment deposition as well as the paleoenvironmental context of the human settlement in Buran-Kaya III.

### Stable isotope analysis and radiocarbon dating

Bone samples from the 2001 excavations were radiocarbon dated by Accelerator Mass Spectrometry (AMS) using purified collagen. These analyses were performed at the Center for Isotope Research of Groningen University, Netherlands (GrA), and at the Laboratoire des Sciences du Climat et de l'Environnement (LSCE) and on Artemis at the Laboratoire de Mesure du C-14 (LMC-14, Commissariat à l'Énergie Atomique, Saclay/Gif-sur-Yvette, France) (GifA/SacA).

The AMS ^14^C dating of human bone was performed from layer 6-1. A red deer specimen (metacarpal, *Cervus elaphus*) from the same layer has been also dated. One horse sample (metatarsal, *Equus* sp.) from layer 6-2 was dated at both the Groningen and Gif-sur-Yvette/Saclay laboratories. The dates were calibrated using the software CalibREV6.0.0 based on the IntCal09 calibration dataset [44].

Collagen extraction from the Buran-Kaya III samples was performed following a protocol adapted from Longin [83] at the Center for Isotope Research of Groningen (human bone) and at the Center for Archaeological Science, University of Tübingen (red deer and horse). At the LSCE, bones were prepared by the method proposed by Nelson [84] based on chemical reaction between ninhydrin and the amino-acids of collagen [85].

Measurements of stable isotopes were performed on a continuous-flow isotope-ratio mass spectrometer (Thermo Quest Delta+XL) connected to a NC 2500 elemental analyzer at the Institute of Geoscience, University of Tübingen, Germany.

Isotopic abundances are expressed as δ values as follows: δX = ((R_sample_/R_standard_)-1)*1000 ‰, where X stands for ^13^C or ^15^N and R is the isotopic ratio ^13^C/^12^C or ^15^N/^14^N. The calibration was based on USGS 24 (δ^13^C = −16.0‰ relatively to the international standard VPDB) for δ^13^C and AEA 305 A (δ^15^N = 39.8‰, relatively to the international standard AIR) for δ^15^N. The analytical error, based on replicate measurements of modern collagen, was ±0.1‰ for δ^13^C values and ±0.2‰ for δ^15^N values. The chemical composition of the collagen is used to assess the reliability of the isotopic measurements. We determined the preservation of the bone collagen using the C/N atomic ratio. Carbon and nitrogen contents (C% and N%) from the collagen of the samples are comparable to those found in modern mammals.

### Zooarcheological analyses

The zooarcheological analyses were conducted on the whole faunal assemblage of large mammals: taxonomical and anatomical determination, taphonomical analyses (following the methodology of Villa [63], Boulestin [86], Lyman [87], Patou-Mathis [88]). Remains were counted using the following units: Number of Identified Specimens (NISP), Combined Minimum Number of Individuals (cMNI), Percentage of element Representation (PR or % survival) defined as PR(i) = (MNE(i)*100)/(N(i)*cMNI); MNE = Minimum Number of Elements and N(i) is defined as the number of time that one element (i) occurs in one skeleton). Paleoecological information was obtained from the distribution of large mammal associations [89].

### Anthropological analyses

Regarding the anthropological analyses, the MNI (Minimum Number of Individuals) was based on the dental wear status, the estimation of the dental development, the position of the enamel hypoplasia and the frequency of each tooth. The sex assessment of these individuals was not possible due to the absence of the pelvic bone, which is a good estimator of sex determination [90]. The determination of the dental developmental age stages (juvenile, sub-adult, adult) was based on the degree of occlusal wear and the dental development (cusp and root developments).

The taxonomic assignment was made only on a few complete bones and complete teeth. The dental non-metric traits have been recorded using the Arizona State University Dental Anthropology System (ASUDAS) [91], [92] and described in Bailey's publications [12], [55], [93].

Areas with striations were replicated by using Coltene President light body silicone moulds. These were subsequently studied under a stereomicroscope and an Environmental Scanning Electron Microscope (ESEM) at the Muséum National d'Histoire Naturelle de Paris and at the Laboratoire de Micropaléontologie, Université Paris 6, Pierre et Marie Curie, UPMC.
